# Identification of Novel miRNAs Involved in Cardiac Repair Following Infarction in Fetal and Adolescent Sheep Hearts

**DOI:** 10.3389/fphys.2020.00614

**Published:** 2020-06-10

**Authors:** Mitchell C. Lock, Ross L. Tellam, Jack R. T. Darby, Jia Yin Soo, Doug A. Brooks, Mike Seed, Joseph B. Selvanayagam, Janna L. Morrison

**Affiliations:** ^1^Early Origins of Adult Health Research Group, University of South Australia, Adelaide, SA, Australia; ^2^Mechanisms in Cell Biology and Disease Research Group, School of Pharmacy and Medical Sciences, University of South Australia, Adelaide, SA, Australia; ^3^Division of Cardiology, The Hospital for Sick Children, Toronto, ON, Canada; ^4^Cardiac Imaging Research, Department of Heart Health, South Australian Health & Medical Research Institute, Flinders University, Adelaide, SA, Australia

**Keywords:** cardiac, regeneration, fetus, myocardial infarction, miRNA

## Abstract

**Aims:**

Animal models have been used to show that there are critical molecular mechanisms that can be activated to induce myocardial repair at specific times in development. For example, specific miRNAs are critical for regulating the response to myocardial infarction (MI) and improving the response to injury. Manipulating these miRNAs in small animal models provides beneficial effects post-MI; however it is not known if these miRNAs are regulated similarly in large mammals. Studying a large animal where the timing of heart development in relation to birth is similar to humans may provide insights to better understand the capacity to repair a developing mammalian heart and its application to the adult heart.

**Methods:**

We used a sheep model of MI that included permanent ligation of the left anterior descending (LAD) coronary artery. Surgery was performed on fetuses (at 105 days gestation when all cardiomyocytes are mononucleated and proliferative) and adolescent sheep (at 6 months of age when all cardiomyocytes contribute to heart growth by hypertrophy). A microarray was utilized to determine the expression of known miRNAs within the damaged and undamaged tissue regions in fetal and adolescent hearts after MI.

**Results:**

73 miRNAs were up-regulated and 58 miRNAs were down-regulated significantly within the fetal infarct compared to remote cardiac samples. From adolescent hearts 69 non-redundant miRNAs were up-regulated and 63 miRNAs were down-regulated significantly in the infarct area compared to remote samples. Opposite differential expression profiles of 10 miRNAs within tissue regions (Infarct area, Border zone and Remote area of the left ventricle) occurred between the fetuses and adolescent sheep. These included miR-558 and miR-1538, which when suppressed using LNA anti-miRNAs in cell culture, increased cardiomyoblast proliferation.

**Conclusion:**

There were significant differences in miRNA responses in fetal and adolescent sheep hearts following a MI, suggesting that the modulation of novel miRNA expression may have therapeutic potential, by promoting proliferation or repair in a damaged heart.

## Introduction

Cardiovascular disease is one of the largest causes of morbidity and mortality worldwide, and is due to the limited capacity to repair human adult heart tissue after myocardial damage (Wong et al., 2012; Benjamin et al., 2017; Wilkins et al., 2017). The lack of regenerative potential is presumed to be the result of cardiomyocyte quiescence, and although there is some evidence for cardiomyocyte proliferation in adult humans, this is insufficient for regeneration or repair (Bergmann et al., 2009; Barnett and van den Hoff, 2011). Current treatments after acute coronary syndromes address the ongoing symptoms and attempt to prevent re-infarction (Dalal et al., 2015). The current lack of effective treatments for repairing heart tissue damage necessitate new approaches to promote the regeneration of adult human heart tissue.

The adult zebrafish, neonatal mouse and fetal sheep have a remarkable ability to regenerate heart tissue after myocardial infarction (MI) (Herdrich et al., 2010; Jopling et al., 2010; Porrello et al., 2011). By studying the transitional period of cardiomyocyte proliferation to quiescence in zebrafish and mouse models, several promising target miRNAs are associated with regeneration For example, the inhibition of miR-15 family members leads to increased mitosis of cardiomyocytes in neonatal mice, promotes adult mouse cardiomyocyte proliferation, improves cardiac function after MI and improves contractile function after ischemia/reperfusion injury (Hullinger et al., 2012; Porrello et al., 2012). miR-133 is down-regulated during the period of cardiomyocyte regeneration and proliferation in injured zebrafish myocardium, implicating it in the regulation of cell cycle progression (Yin et al., 2012). The inhibition of miR-34 family members has shown therapeutic potential, where cardiac remodeling was attenuated and improved cardiac function in mouse models of both pressure overload and MI with up-regulation of growth factor target genes including *Vegf, Vcl, Sirt1, Notch1*, and *Pofut1* (Bernardo et al., 2012; Boon et al., 2013). Lastly, inhibition of target genes of miR-590 and miR-199a, such as *Clic5, Hopx*, and *Homer1*, using short interfering RNAs, results in approximately double the number of cardiomyocytes undergoing DNA synthesis and significantly increased cytokinesis (Eulalio et al., 2012). Studying a model organism for heart regeneration that closely reflects the cardiac developmental timing of humans, such as sheep (Botting et al., 2012; Lock et al., 2017, 2018; Morrison et al., 2018), may reveal additional miRNAs that regulate cardiomyocyte proliferation, and which may potentially have clinical relevance. We therefore utilized a sheep model of MI to investigate the differential miRNA expression in regenerative fetal hearts and non-regenerative adolescent hearts.

## Materials and Methods

### Animal Ethics and Housing

Experimental protocols for animal research were approved by the South Australian Health and Medical Research Institute (SAHMRI) Animal Ethics Committee (SAM046). Experiments were designed and reported with reference to the ARRIVE guidelines (Kilkenny et al., 2010). The experiments comply with the policies and regulations of the European Convention for the Protection of Vertebrate Animals used for Experimental and other Scientific Purposes (Grundy, 2015). In total, 10 Merino ewes and their fetuses and 12 adolescent sheep (∼6 months old) were used in this study (supplied by South Australian Medical Science Research Institute). Each ewe or adolescent sheep was housed in an individual pen in an indoor housing facility (Preclinical Imaging and Research Laboratories, SAHMRI) that was maintained at a constant ambient temperature of between 20 and 22°C with a 12 h light/dark cycle.

### Surgical Procedure to Ligate the Left Anterior Descending (LAD) Coronary Artery

At 102 days gestation (term, 150 days), ewes underwent surgery under aseptic conditions using general anaesthesia induced by the intravenous infusion of diazepam (0.3 mg/kg) and ketamine (7 mg/kg), and maintained with inhalation of isoflurane (1–2%) in oxygen. Briefly, vascular catheters (Critchley Electrical Products, Silverwater, Australia) were inserted as previously described (Danielson et al., 2005; Duan et al., 2017; Lock et al., 2020) in the maternal jugular vein, the amniotic cavity and the fetal carotid artery and jugular vein.

Animals underwent thoracotomy and were randomly allocated to Sham surgery (fetus, *n* = 5; adolescent sheep, *n* = 5) or ligation of the left anterior descending (LAD) coronary artery (fetus, *n* = 5; adolescent sheep, *n* = 5) to induce infarction. Lignocaine was administered intravenously to all fetuses (0.2 mL bolus) and adolescents (100 mg/500 mL) prior to incising the pericardial sac. For animals in the infarction group, a silk suture was placed around the second diagonal of the LAD coronary artery and tied off, while observing blanching of the heart tissue. The thoracotomy incision was tightly sutured in layers (ribs, muscle and skin). The fetus was then returned to the ewe’s uterus and the uterus was sutured closed.

Fetal catheters were exteriorized through a small incision in the ewe’s flank. At surgery, antibiotics were administered to the ewe (154 mg of Procaine penicillin, 393 mg of benzathine penicillin, 500 mg of dihydrostreptomycin; Lyppards, Adelaide, Australia) and fetuses (150 mg of Procaine penicillin, 112 mg of benzathine penicillin, 250 mg of dihydrostreptomycin; Lyppards). When the ewes and adolescent sheep recovered from anesthesia, they were given analgesia (20 μg/kg, Xylazil, Troy Laboratories, Australia). Antibiotics were administered intramuscularly to each ewe or adolescent sheep for 3 days after surgery and to each fetus intra-amniotically (500 mg of ampicillin; Lyppards).

### Post-mortem and Tissue Collection

On the 3rd day after ligation of the LAD coronary artery ewes and adolescent sheep were humanely killed via overdose of sodium pentobarbitone (8 g; Vibrac Australia, Peakhurst, Australia). The ewes’ uterus was removed by hysterotomy, and the fetus was removed and weighed. The heart was quickly dissected, weighed and reverse perfused through the aorta with heparin sulfate (5 mL; to prevent clotting and flush blood from the heart) and a saturated KCl solution (5 mL; to arrest the heart in diastole). The heart was photographed, cut into sections and the infarct visualized using 2,3,5-triphenyltetrazolium chloride (TTC) staining (as previously published; Duan et al., 2017; Lock et al., 2019). A total of 10 fetuses [Sham, *n* = 5 (3 female, 2 male); MI, *n* = 5 (3 male, 2 female)] and 10 adolescent sheep (Sham, *n* = 5; MI, *n* = 5 all male) underwent post-mortem and were used for molecular analyses. Ventricle tissue was collected from the infarct area, the border zone (salvageable tissue immediately surrounding the Infarct area) and a remote area of the left ventricle, as well as the corresponding areas from Sham animals. Tissue was either frozen in liquid nitrogen for miRNA microarray and qRT-PCR analyses or fixed in 4% paraformaldehyde for histological and immunohistochemistry analyses.

### miRNA Microarray Analysis

A custom designed miRNA microarray was employed using a service provider (LC Sciences, United States; Morrison et al., 2015). It contained multiple (3–8) replicates of 3,098 probes and multiple replicates (8–80) of 56 control probes. The former probes were for identified ovine miRNA and additional mammalian miRNA sequences downloaded from miRBase^1^, as previously described (Morrison et al., 2015). The available miRBase entries for ovine species are limited; however, since many miRNAs are highly conserved amongst species, the microarray was supplemented with miRNAs from other species. The multispecies probes on the microarray caused redundancy by design. The experimental design included three biological replicates from each treatment group and age.

Background subtracted raw data from the manufacturer (LC Sciences, United States; Casella and Berger, 2001; Bolstad, 2004) was analyzed using a 2-factor ANOVA (factors: age (Fetus and Adolescent) and region (Remote, Border and Infarct); 6 groups) (Eisen et al., 1998). Data was filtered at *P* < 0.05 for each factor and the interaction. For miRNA probes identified as significant for an interaction, one-way ANOVA was used to determine significantly deregulated probes within each age group. *P*-values for this analysis were not corrected for multiple testing, as although there were 1894 expressed probes, many of these were for 808 unique miRNAs, with some probes likely to also cross hybridize or reflect highly related miRNA families. Thus, many of the probes on the microarray were not independent. For each factor and interaction, the mean value of all probes was taken for the six groupings (Fetal and Adolescent, Remote, Border, and Infarct). Probes with overall mean signal less than 500 (unreliable signal) as well as any probes with a signal of zero in one or more sample (unreliable probes) were then removed. K-means clustering was performed using the CLCBIO Genomics Workbench program suite^2^ using the mean probe values from each group. Data reported in the clustering analysis are represented as untransformed raw signal. Euclidean distance and *K* = 10 were selected for k-means clustering as this revealed profile stability with the addition of further clusters not creating any substantially different patterns. Unsupervised principal component analysis (PCA) and hierarchical clustering analyses of sample data used the default options in ClustVis^3^.

### miRNA Target Prediction

Significantly deregulated probes were separated into groups based on their expression profiles across tissue regions at both ages. miRNA target predictions for the significantly up-regulated or down-regulated probes in the Infarct samples compared to the Remote samples was performed using miRWalk 3 (University of Heidelberg^4^). miRNA target prediction involves high rates of false positive targets and hence conservative strategies were utilized (Pinzon et al., 2017). MiRWalk used a consensus approach based on the intersection of at least three different methods and utilized target mRNA information from the human, bovine, and mouse genomes.

### Predicted Target Term Enrichments

Gene Ontology (GO) term enrichments and KEGG pathway functional enrichments were performed using miRWalk 3 by hypergeometric tests (Fisher-exact-test). Redundant terms generated by the multiple species background were removed. Only enriched terms with an FDR corrected *P* < 0.05 were used.

### Real-Time PCR for miRNA and Target Genes

All essential information regarding the qRT-PCR procedure is included as per the MIQE guidelines (Bustin et al., 2009). Total RNA was extracted from frozen heart tissue for each fetus and adolescent sheep using QIAzol Lysis Reagent solution and QIAgen miRNeasy purification columns, as per manufacturer guidelines (Qiagen, Germany). Total RNA was quantified by spectrophotometric measurements at 260 and 280 nm in a NanoDrop Lite Spectrophotometer (Thermo Fisher Scientific) and the 260/280 nm ratio. RNA samples were checked for integrity, as well as protein and DNA contamination using spectrophotometer results and agarose gel stained using ethidium bromide. cDNA was synthesized using Superscript III First Strand Synthesis System (Invitrogen, United States) using 1 μg of total RNA, random hexamers, dNTP, DTT and Superscript III in a final volume of 20 μL, as per the manufacturer’s guidelines in a MJ Mini personal thermocycler (Biorad, United States). Controls containing either no RNA transcript or no Superscript III were used to test for reagent contamination and genomic DNA contamination, respectively. miRNA cDNA was synthesized using the miScript II RT Kit (Qiagen, Germany) according to the manufacturer’s guidelines. Each sample contained 4 μL 5x miScript Hiflex buffer, 2 μL 10x Nucleics mix, 2 μL miScript Reverse Transcriptase mix and 1 μg extracted RNA. The no amplification control (NAC) negative control samples replaced the miScript Reverse Transcriptase mix with 2 μL of molecular grade water. The geNorm component of qbaseplus 2.0 software (Biogazelle, Belgium) was used to determine the most stable reference genes from a panel of candidate reference genes (Vandesompele et al., 2002) and the minimum number of reference genes required to calculate a stable normalization factor, as previously described (Soo et al., 2012; McGillick et al., 2013). For qRT-PCR data output normalization, three stable reference genes *RPLP0* (NM_001012682.1), *HPRT1* (NM_001034035.1) and *YWHAZ* (AY970970) (Passmore et al., 2009) were run in parallel with all target genes, as previously described. For miRNA qRT-PCR, target miRNAs were normalized against miR-208 (MI0000251); miR-92-1 (MS00006594) and SNORD61-1 (MS00033705, QIAGEN, Australia). Relative expression of target genes (*BIRC5*, NM_001001855.2; *SPAG5*, XM_004012500.1; *CHEK1*, XM_004019518.1; *CDK1*, NM_001142509.1; *GJA1*, XM_004011159.1; *CTGF*, NM_001164714.1; *PGAM1*, NM_001034054; *SRF*, XM_004019222.1; *CLIC5*, XM_004018860.1; *HOPX*, NM_174097.2, and *HOMER1*, NM_001076052.1) and miRNAs (miR-15a, MS00008785; miR-15b, MS00008799; miR-16, MS00031493; miR-195, MS00008953; miR497, MS00031906; miR-199a, MS00007602; and miR-590, MS00010269; QIAGEN, Australia) were measured by qRT-PCR using KiCqStart SYBR Green qPCR ReadyMix (Sigma Aldrich, United States) or miScript SYBR Green PCR Kit (QIAGEN, Australia) in a final volume of 6 μL on a ViiA7 Fast Real-time PCR system (Applied Biosystems, United States) as previously described. Each qRT-PCR well contained 3 μL SYBR Green Master Mix (2X), 2 μL of forward and reverse primer mixed with H_2_O to obtain final primer concentrations and 1 μL of diluted relevant cDNA. Each sample was run in triplicate for target genes and reference genes. The abundance of each transcript relative to the abundance of stable reference genes (Hellemans et al., 2007) was calculated using DataAssist 3.0 analysis software (Applied Biosystems, United States) and expressed as mRNA mean normalized expression (MNE) ± SEM. Outliers were identified using the ROUT method with a false discovery rate *Q* = 1% (GraphPad Prism 8, United States) and were removed from the analysis.

### H9c2 Cell Culture Experiments

H9c2 cells derived from rat embryonic ventricular cardiomyoblasts were cultured in Glutamax DMEM (Gibco, United States) media that contained 10% (v/v) fetal bovine serum and 1% v/v penicillin/streptomycin at 37°C in an atmosphere of 95% air and 5% CO_2_. Hypoxia was induced by incubation in sealed hypoxia chambers containing 94% N_2_, 5% CO_2_, and 1% O_2_ to simulate tissue hypoxia during ischemia (Yao et al., 2017). Since miRNAs are generally conserved between species, we first checked that the sequences of the chosen miRNAs were not different between species using miRbase (University of Manchester^1^) and used the HSA version of the miRNA for the remainder of the experiments to improve interspecies translation. Cells were seeded into 6-well-plates at a density of 100,000 cells per well and 96-well-plates at 5000 cells per well. 100 nmol miRNA-inhibitors [hsa-miR-558, hsa-miR-1538, hsa-miR-150 or the manufacturer negative control 5 (NC5) miRNA (IDT, United States)] were each diluted in 125 μL OPTIMEM media (Gibco, United States) and combined with Lipofectamine 2000 (Thermo Fisher Scientific, United States) before further dilution in culture media. Cells were treated with either normal media Glucose/Normoxia, No-Glucose/Normoxia, Glucose/Hypoxia or No-Glucose/Hypoxia culture conditions for 48 h to simulate lack of oxygen and glucose supply during an infarction. Cells in 6-well-plates were either lysed in Qiazol (Qiagen, United States) for qRT-PCR or fixed in 4% formaldehyde (Sigma Aldrich, United States) for subsequent immunohistochemistry analysis. 96-well-plates were aspirated of culture media after treatment and 100 μL fresh media was added to each well. Twenty microliter of MTS Proliferation assay reagent (Promega, United States) was then added to each well and incubated for 2 h at 37°C. Thereafter the absorption of each well was measured at the wavelength of 490 nm by a spectrophotometer. Potential target genes of novel miRNAs were determined using miRWalk 3 (University of Heidelberg^4^). Target genes were selected based on how significantly likely they were to be modulated by the miRNAs of interest and are expressed in cardiac tissue. The expression of target genes *PAPPA, JAG, NF2, MYOC1*, and *SOX4* act as markers to ensure that miRNA inhibition is effectively associated an upregulation of target gene expression.

### Immunohistochemistry

Rehydrated cardiomyoblasts were blocked for endogenous peroxidase activity with 3% hydrogen peroxide (Sigma-Aldrich, United States), followed by heat-induced antigen retrieval in sodium-citrate buffer (pH 6.0). Slides were incubated overnight with the primary antibody (Aurora-B, ab2254, Abcam, United States) at 4°C following incubation with non-immune serum (serum-blocking solution; Histostain-Plus Kit; Invitrogen, United States) to prevent non-specific binding. Negative control slides with the primary antibody omitted were used to demonstrate the absence of non-specific binding of the secondary antibody or reagent contamination. In addition, negative control slides, where primary antibody was substituted by rabbit serum (Sigma-Aldrich, United States) were carried out at the same protein concentration as the diluted primary antibodies (Hewitt et al., 2014). Negative controls were incubated overnight at 4°C in parallel with test slides under the same experimental conditions. A Histostain-Plus kit (Invitrogen, United States) was used with horseradish peroxidase and Histostain-Plus broad spectrum 3,3’-diaminobenzidine (DAB) chromagen for visualization of positive cells. All sections were counterstained with Mayer’s hematoxylin (Sigma-Aldrich, United States). Following optimization, the substrate-chromagen reaction was allowed to occur for the same time for all slides. Images of stained slides were taken using a NanoZoomer-XR (Hamamatsu, Japan) and quantified using the VIS software package (Visiopharm, Denmark), as previously described (Lock et al., 2015).

### Statistical Analysis

Statistical analyses for qRT-PCR and immunohistochemistry were performed within the STATA 12 program (StataCorp, United States). Analyses between tissue regions (Infarct vs. Border vs. Remote) at each age were assessed using a nested Analysis of Variance (ANOVA). A Bonferroni *post hoc* test was performed with multiple comparisons for each tissue region against the Sham tissue.

## Results

Two strategies were employed to assess the expression of miRNAs after MI in the fetus and adolescent sheep heart samples. qRT-PCR was used to evaluate changes in the expression of key miRNAs and their target genes, which have been identified as playing important roles in cardiac regeneration in zebrafish and mouse models. miRNA microarray analysis provided a more comprehensive analysis to profile 3,098 probes representing miRNAs from several mammalian species, to then identify differentially expressed probes, and to predict mRNA targets and enriched functions associated with clustered unique miRNAs.

### Profiling of Zebrafish and Mouse miRNAs Implicated in Cardiac Regeneration in Sheep

A number of miRNAs have been identified as possible therapeutic targets in small animals, which retain heart regenerative capacity for a period of time after birth. The most promising of these candidate miRNAs were investigated in our large animal model to determine if the changes in miRNA expression are consistent across species.

#### miR-15 Family

**FIGURE 1:**
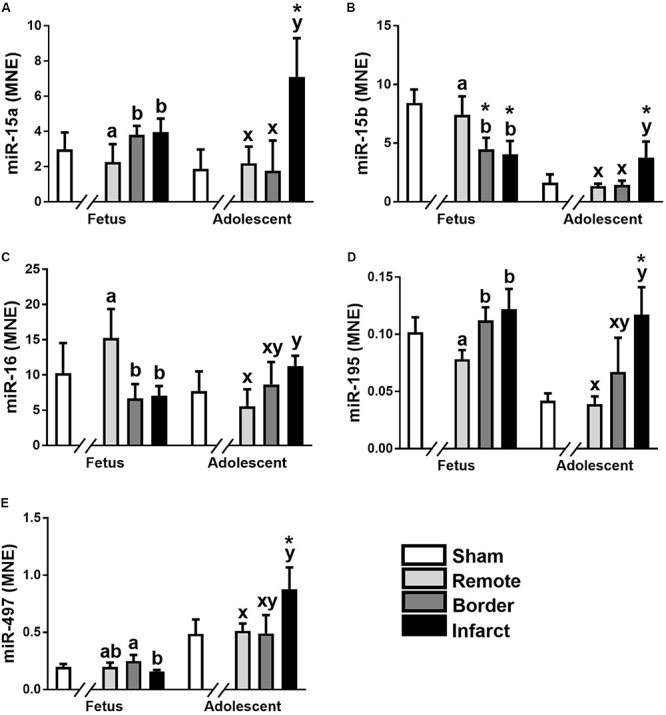
Expression of the miR-15 family in sheep 3 days post-MI. Mean normalized expression (MNE) of miR-15a **(A)**, miR-15b **(B)**, miR-16 **(C)**, miR-195 **(D),** and miR-497 **(E)** in Sham, Remote, Border zone, and Infarct Tissue. Superscript letters (Fetal Sheep; a, b and Adolescent Sheep; x, y) represent significance between tissue regions (Remote, Border and Infarct) at each age (*P* < 0.05). *Represents significantly different data from the sham animals at each age (*P* < 0.05). Analyses between tissue regions (Infarct vs. Border vs. Remote) at each age were assessed using a nested Analysis of variance (ANOVA). A Bonferroni *post hoc* test was performed with multiple comparisons for each tissue region against the Sham tissue. *n* = 5 per treatment group per age.

**FIGURE 2:**
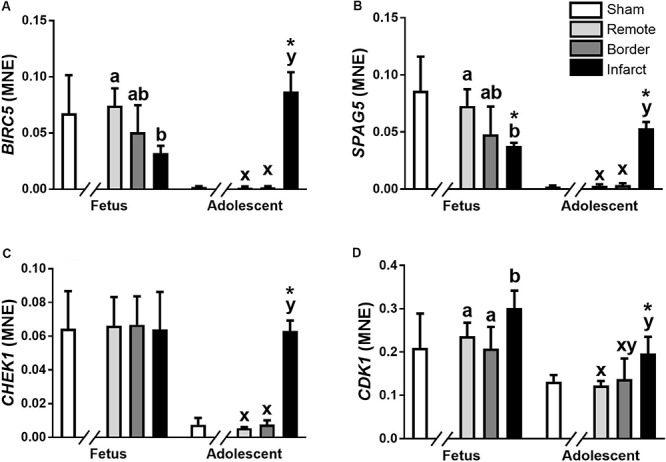
qRT-PCR validation of miR-15 family target mRNA expression in sheep 3 days post-MI. Mean normalized expression (MNE) of *BIRC5* **(A)**, *SPAG5* **(B)**, *CHEK1* **(C),** and *CDK1* **(D)** in Sham, Remote, Border zone, and Infarct Tissue. Superscript letters (Fetal Sheep; a, b and Adolescent Sheep; x, y) represent significance between tissue regions (Remote, Border and Infarct) at each age (*P* < 0.05). *Represents significantly different data from the sham animals at each age (*P* < 0.05). Analyses between tissue regions (Infarct vs. Border vs. Remote) at each age were assessed using a nested Analysis of Variance (ANOVA). A Bonferroni *post hoc* test was performed with multiple comparisons for each tissue region against the Sham tissue. *n* = 5 per treatment group per age.

The miR-15 family contains five miRNA, miR-15a, miR-15b, miR-16, miR-195, and miR-497. In the ventricular tissue from adolescent animals there was a significant increase in expression of miR-15a in the Infarct sample compared to the Remote samples and Sham controls (Figure 1A; *P* = 0.017, 0.0017, respectively). miR-15b was decreased in the Border and Infarct samples compared to both the Remote samples and Sham controls in the fetuses (Figure 1B; *P* ≤ 0.05, < 0.001, respectively). In the adolescent sheep, there was an increase in miR-15b expression in the Infarct samples compared to Sham controls, as well as Remote and Border samples (Figure 1B; *P* = 0.007, *P* < 0.001, respectively). The expression of miR-16 was decreased in the fetal Border and Infarct samples compared with the Remote sample (*P* < 0.05) and was increased in the Infarct sample compared with Remote samples (*P* < 0.05) in the adolescent sheep (Figure 1C). miR-195 expression was increased in the fetal Border and Infarct samples compared to Remote samples (Figure 1D; *P* < 0.05). In the adolescent animals there was an increase in expression of miR-195 in the Infarct samples compared to Remote and Sham samples (Figure 1D; *P* < 0.05, *P* < 0.001 respectively). The expression of miR-497 was significantly decreased in the fetal Infarct samples compared to Border samples (Figure 1E; *P* < 0.05). In the adolescent sheep there was an increase in miR-497 expression in the Infarct samples compared to Remote and Sham samples (Figure 1E, *P* < 0.05, *P* = 0.007 respectively). miR-15 family target genes demonstrated similar expression profiles (Figure 2).

#### miR-133a

**FIGURE 3:**
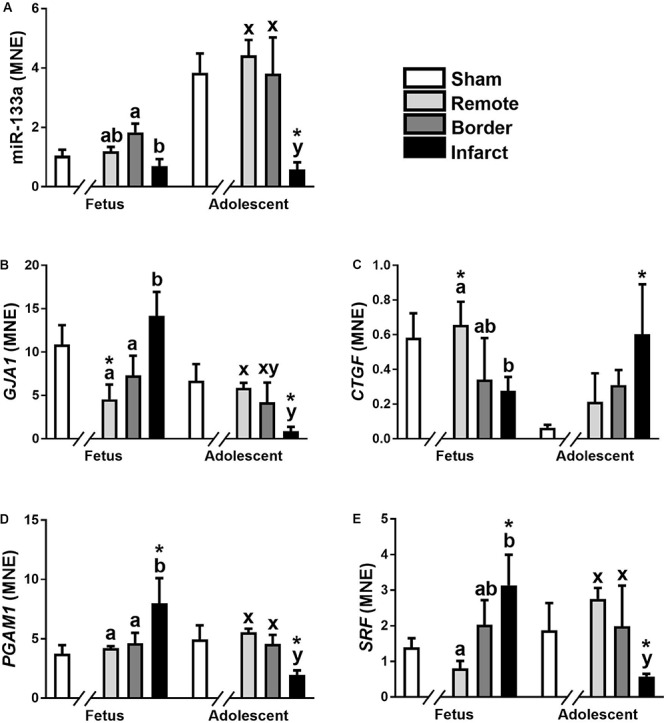
qRT-PCR validation of miR-133a and mRNA target expression in sheep 3 days post-MI. Mean normalized expression (MNE) of miR-133a **(A)**, *GJA1* **(B)**, *CTGF* **(C)**, *PGAM1* **(D)**, and *SRF* **(E)** in Sham, Remote, Border zone, and Infarct Tissue. Superscript letters (Fetal Sheep; a, b and Adolescent Sheep; x, y) represent significance between tissue regions (Remote, Border, and Infarct) at each age (*P* < 0.05). *Represents significantly different data from the sham animals at each age (*P* < 0.05). Analyses between tissue regions (Infarct vs. Border vs. Remote) at each age were assessed using a nested Analysis of variance (ANOVA). A Bonferroni *post hoc* test was performed with multiple comparisons for each tissue region against the Sham tissue. *n* = 5 per treatment group per age.

The expression of miR-133a was significantly down-regulated in fetal Infarct compared to Border samples (Figure 3A; *P* < 0.05). In adolescent sheep, there was a significant decrease in Infarct compared to Remote, Border and Sham samples (Figure 3A; *P* < 0.001, *P* < 0.05, *P* < 0.001, respectively). The higher expression of this miRNA in adolescent sheep is consistent with increased expression of miR-133a with age in multiple species (Liu et al., 2008; Morrison et al., 2015); but, the decreased expression in the Infarct samples within adolescent sheep was unexpected. miR-133a target gene expression did not mirror the expression of miR-133a (Figures 3B–E).

#### miR-34 Family

miR-34a expression was significantly higher in the adolescent sheep heart samples compared to the fetuses (*P* < 0.0001, Supplementary Files). There was no significant difference in the expression of miR-34a in the fetal Infarct samples after MI; but, miR-34awas down-regulated in the adolescent Infarct samples compared to Remote samples (*P* = 0.018, Supplementary Files). miR-34b expression was not significantly different at either age.

#### miR-25

miR-25 is another miRNA that has a reported role in cardio-protection after infarction. Interestingly, in the current investigation miR-25 was not significantly changed in the fetal heart in response to MI, but this miRNA was up-regulated in the Infarct samples in the adolescent animals compared to Remote samples (*P* < 0.004, Supplementary Files).

#### miR-199a

miR-199a and miR-590 have cardioprotective roles after infarction in mice (Eulalio et al., 2012; Ali, 2013; Wang and Martin, 2014). miR-199a was significantly up-regulated in the Infarct samples compared to the Remote samples in both fetuses and adolescent sheep (*P* = 0.009, *P* = 0.007, respectively, Supplementary Files). However, their target genes were up-regulated in the infarct area compared to Remote and Sham samples, indicating that these miRNAs have a smaller role in sheep cardiac regeneration (Supplementary Figure 1).

#### miR-17∼miR-92 Cluster

The miR-17∼miR-92 cluster of miRNAs are also potential promoters of cell cycle in mice and sheep that are down-regulated at birth (Chen et al., 2013; Morrison et al., 2015). miR-17, miR-18a and miR-92a expression were significantly higher in the fetuses compared to adolescent sheep (*P* = 0.01, 0.004, 0.02, respectively, Supplementary Files); however, there was no effect of MI on expression at either age. Expression of miR-19a was too low for statistical analysis, but miR-19b was highly expressed in fetuses, although expression remained unchanged between the MI tissue regions. However, miR-19b was significantly up-regulated in the adolescent sheep in the Infarct samples compared to Remote samples (*P* = 0.03, Supplementary Files). Expression of miR-20a and miR-20b expression were higher in the fetuses compared to the adolescent animals (*P* = 0.03, *P* = 0.007, respectively, Supplementary Files), but were unchanged at both ages in response to MI.

### miRNA Microarray Analysis

#### miRNA Probe PCA Analysis and Clustering

**FIGURE 4:**
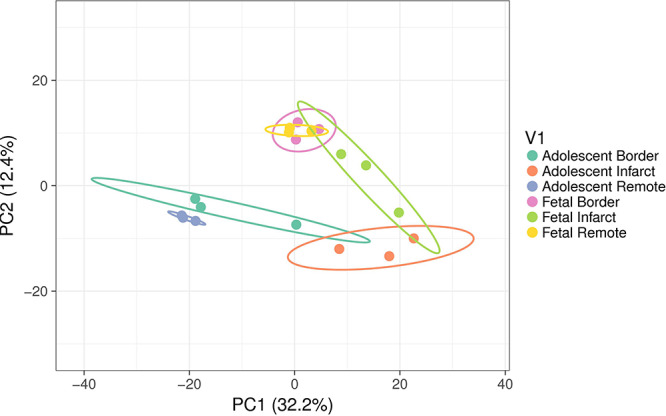
Principal component analysis (PCA) of miRNA expression using all samples. The ellipses show the 95% confidence level for each group. Each group consisted of samples from three animals. The variance explained in each principal component (PC) is shown in brackets. The PC2 dimension separated the groups on the basis of age while separation of groups along PC1 highlighted the infarction response for both ages. The fetal Remote and Border samples were near superimposable. The adolescent Border sample showed considerable variance along PC1.

miRNA microarrays were used to investigate the immediate miRNA response to infarction [*n* = 3 per tissue region (Infarct, Border, Remote) and at each age (Fetal, Adolescent)]. This approach complements the qRT-PCR targeted miRNA approach and has the potential to identify additional miRNAs and broad functional themes associated with cardiac regeneration in sheep. A PCA analysis of the microarray data shows separation of the two age groups and three treatment groups as well as clustering of the biological replicates within each of the groups (Figure 4). The PC2 dimension separated the groups on the basis of age, while separation of groups along PC1 highlighted the infarction response for both ages. The fetal Remote and Border samples were nearly superimposable. Overall, the fetal response to infarction was more constrained than the adolescent response. The adolescent Border sample showed considerable variance along PC1. The analysis demonstrates that multiple miRNAs have altered expression in the response to cardiac damage in the sheep; this suggests substantial complexity in the miRNA regulation of the heart regenerative response.

K-means clustering (*k* = 10) of the significant differentially expressed (DE) probes was undertaken separately at each age. This analysis clusters together miRNAs with similar expression changes in response to MI (Table 1 and Supplementary Figures 2, 3). Clusters 7, 8, and 9 in the adolescent animals were combined for mRNA target prediction as they contained multispecies probes for the same miRNAs. Cluster 1 in the fetal samples had insufficient mRNA targets for gene ontology analysis. Clusters 3 and 6 in the fetuses and Cluster 5 in adolescent sheep were the largest, containing 41, 46, and 58 probes respectively. Cluster 3 in the fetal samples showed an overall increase in expression in Infarct compared to Remote samples, while Cluster 6 showed an overall decline in miRNA expression in the Infarct samples compared to the Remote samples. In the fetal samples, Clusters 1, 2, 3, 4, and 5 were all increasing expression in the Infarct samples compared with Remote samples. Clusters 6, 7, 9, and 10 all had declining expression in the Infarct samples compared with Remote samples. In the adolescent sheep Clusters 1, 3, 4, 6, 7, 9, and 10 all had increased expression in the Infarct samples compared with Remote samples. Clusters 2, 5, and 8 were all declining expression in the Infarct samples compared with Remote samples.

**TABLE 1:** miRNA expression k-means clusters.

**Fetal cluster number**	**Direction of change**	**miRNA**
1	Increased in infarct	miR-21, miR-21a-5p, miR-451, miR-7641
2	Increased in infarct	let-7j, miR-1273e, miR-1285, miR-155, miR-199a-3p, miR-21, miR-222a-3p, miR-223-3p, miR-2478, miR-25, miR-25-3p, miR-29a, miR-376c-3p, miR-381, miR-381-5p, miR-3957-3p, miR-451, miR-451b, miR-5100, miR-6236, miR-6240, miR-7977
3	Increased in infarct	miR-106a, miR-1224-5p, miR-1246, miR-125b, miR-125b-5p, miR-1260, miR-1260a, miR-1260b, miR-1285, miR-1357, miR-1386, miR-142-3p, **miR-15a**, **miR-16a**, miR-1895, miR-199b, miR-19b, miR-20a, miR-20b, miR-21, miR-223, miR-2311, miR-2478, miR-2887, miR-2888, miR-29a-3p, miR-29d-3p, miR-30b-3p, miR-339b, miR-379-5p, miR-3968, miR-4454, miR-4792, miR-486-5p, miR-5100, miR-6119-5p, miR-6240, miR-6412, miR-716a, miR-716b, miR-7641
4	Increased in infarct	let-7i, miR-1246, miR-1254, miR-127, miR-1273e, miR-1285, miR-140, **miR-16c-5p**, miR-199a-3p, miR-199a-5p, miR-199c, miR-21, miR-2137, miR-214, miR-221, miR-222, miR-30c, miR-451, miR-5126, miR-6323, miR-6236-p5
5	Increased in infarct	miR-451, miR-7641, miR-21
6	Decreased in infarct	let-7e-5p, miR-1, miR-100, miR-1285, **miR-133a-5p**, miR-1386, miR-139, miR-1-3p, miR-150, miR-151-5p, miR-151b, miR-181a, miR-1973, miR-224, miR-23b, miR-23b-3p, miR-24, miR-28c, miR-29a, miR-30a-3p, miR-30c, miR-30d, miR-30d-5p, miR-30e-3p, miR-30f, miR-331-3p, miR-339b, miR-3431, miR-374b, miR-378, miR-378a, miR-378b, miR-378c, miR-378d, miR-378e, miR-378f, miR-378g, miR-378i, miR-4454, miR-452-3p, miR-558, miR-574, miR-6240, miR-669e, miR-7641, miR-99a
7	Decreased in infarct	miR-1, **miR-133**, **miR-133a**, **miR-133a-3p**, miR-133b, miR-133b-3p, miR-374a, miR-499, miR-499-5p, miR-499b-5p
9	Decreased in infarct	miR-133c, miR-145, miR-30a-5p, miR-30b, miR-30b-5p, miR-30c, miR-30c-5p, miR-30d, miR-30e-5p, miR-30f, miR-499
10	Decreased in infarct	miR-125a, miR-125a-5p, miR-1386, miR-1538-p3, miR-181a-5p, miR-181c, miR-2487-p5, miR-30d, miR-4448, miR-4454, miR-5100, miR-6240-p3, miR-716a-p5, miR-716b
**Adolescent cluster number**	**Direction of change**	
1	Increased in infarct	let-7j, miR-1260b, miR-1285, mir-1538, **miR-16c-5p**, miR-21, miR-223, miR-30c-1-3p, miR-3957-3p, miR-4454, miR-5100, miR-5126, mir-6236, mir-6240, mir-716a, miR-716b, miR-7977
2	Decreased in infarct	miR-34a, miR-125b, miR-125b-5p, **miR-133a**, miR-133b-3p, miR-133c, miR-185, miR-197, miR-22-3p, miR-22-5p, miR-23b, miR-24, miR-27b, miR-30a-5p, miR-30b, miR-30b-5p, miR-30c, miR-30c-5p, miR-30d, miR-30d-5p, miR-30e-5p, miR-30f, miR-99a.
3	Increased in infarct	let-7i, miR-106a, miR-106b, miR-1224-5p, miR-1260b, miR-127, miR-1386, miR-150, miR-155, **miR-15a**, miR-1895, miR-199a-3p, miR-199b, miR-19b, miR-20a, miR-20b, miR-2478, miR-25, miR-25-3p, miR-2887, miR-2888, miR-29a, miR-30b-3p, miR-339b, miR-376c-3p, miR-376e-3p, miR-379-5p, miR-3968, miR-409-3p, miR-4454, miR-4792, miR-487b-3p, miR-5100, miR-558, miR-6119-5p, miR-7641
4	Increased in infarct	miR-106a, miR-1246, miR-1260, miR-1260a, miR-1260b, miR-1285, miR-1357, miR-142-3p, **miR-15a-5p**, miR-199a-3p, miR-199a-5p, miR-199c, miR-2137, miR-214, miR-2313-5p, miR-2311, miR-2478, miR-2487, miR-381-5p, miR-4454, miR-4484, miR-451b, miR-5100, miR-6236-5p, miR-6240, miR-6323, miR-6412, miR-716a, miR-716b, miR-7641
5	Decreased in infarct	miR-1, miR-100, miR-103-3p, miR-125a, miR-125a-5p, **miR-133a-5p**, miR-139, miR-1-3p, miR-148b, miR-151-5p, miR-151b, miR-181a, miR-181a-5p, miR-181c, miR-1973, miR-23a-3p, miR-221, miR-222, miR-222a-3p, miR-224, miR-23b-3p, miR-23b, miR-26-5p, miR-28c, miR-29a, miR-29a-3p, miR-29b, miR-29d-3p, miR-2284x, miR-30a-3p, miR-30a-5p, miR-30b, miR-30c, miR-30c-5p, miR-30d-5p, miR-30e-3p, miR-30f, miR-331-3p, miR-339b, miR-3431, miR-361, miR-374b, miR-378, miR-378a, miR-378b, miR-378c, miR-378d, miR-378e, miR-378f, miR-378i, miR-424-5p, miR-452-3p, miR-486, miR-486-5p, miR-4306, miR-574, miR-669e, miR-99a
6	Increased in infarct	miR-1246, miR-1254, miR-1273e, miR-1285, **miR-16a**, miR-21, miR-223-3p, miR-451
7	Increased in infarct	miR-7641
8	Decreased in infarct	miR-1, **miR-133**, **miR-133a**, **miR-133a-3p**, **miR-133a-p5**, miR-133b, miR-499, miR-499-5p, miR-499b-5p, miR-193b
9	Increased in infarct	miR-1273e, miR-21, miR-451, miR-7641
10	Increased in infarct	miR-21, miR-21a-5p, miR-7641

**Differentially expressed microarray probes were first identified and subjected to k-means clustering. All species identifiers were then removed and a non-redundant list of miRNAs was obtained. Anonymous probes beginning with the designation “PC” were excluded. A full list of probes with species identifiers can be found in Supplementary File 1. miRNAs that were included in the targeted analysis of previously implicated miRNAs in mouse and zebrafish studies using qRT-PCR analyses are highlighted in bold.**

### miRNA qRT-PCR Expression Validation

By design, a single miRNA could be separated into multiple clusters due to the array featuring multiple species probes for the same miRNA or probes identifying miRNA family variants. The miRNA expression patterns measured using qRT-PCR were generally consistent with the expression measured by the miRNA microarray. Probes for miR-15a and miR-16 were present in Clusters 3 and 5 in the fetuses and Clusters 1 and 6 in the adolescent sheep. These clusters had increased expression in the Infarct samples compared to Remote samples, which was consistent with qRT-PCR miR-15a expression, whereas miR-16 was down-regulated in fetal Infarct samples in the qRT-PCR data but increased in the microarray data. This latter difference may be explained because the clustered probes for miR-16 were for the miR-16a and miR-16c-5p variants, which were not specifically measured in qRT-PCR. Probes for miR-133a were present in Clusters 6 and 7 in the fetus and Clusters 2, 5, and 8 in the adolescent sheep. These clusters all had decreasing expression profiles that mirrors the qRT-PCR data for miR-133a where there was decreased expression in the Infarct compared with Border samples in the fetal samples and decreased expression in the Infarct compared with the Border and Remote samples in the adolescent sheep. Fold change comparisons (Infarct/Remote) of miRNAs using both methods at each age showed strong correlations with *r*^2^ = 0.91, 0.84, 0.93 and 0.90 (*P* < 0.0001, *P* < 0.0001) for fetus and adolescent samples, respectively (Supplementary Figure 4).

### miRNA Target Prediction and Pathway Enrichment

Microarray probes that were significantly differentially regulated in the Infarct compared to Remote samples were grouped based on expression profile (higher or lower in the Infarct compared to Remote samples at each age group). Since miRNAs have a large number of target mRNAs, and these targets often overlap, there was a high level of consistency between pathways of predicted targets of miRNAs between fetal and adolescent sheep (Supplementary File 2). The most enriched KEGG pathway terms at both ages for the mRNA targets predicted from the significantly deregulated miRNAs included: *Pathways in cancer (map05200)*, *MAPK signaling pathway (map04010)*, *Axon guidance (map04360)*, *Proteoglycans in cancer (map05205)*, *cAMP signaling pathway (map04024)*, *Ras signaling pathway (map04014)*, *Signaling pathways regulating pluripotency of stem cells (map04550)*, *Wnt signaling pathway (map04310)*, *Leukocyte transendothelial migration (map04670)*, *Endocytosis (map04144)* (Supplementary File 2). The most enriched gene ontology terms using Biological Process for the significantly deregulated miRNAs at both ages included: *Canonical Wnt signaling pathway (GO:0060070)*, *Wnt signaling pathway (GO:0016055)*, *Angiogenesis (GO:0001525)*, *Protein ubiquitination (GO:0016567)*, *Positive regulation of GTPase activity (GO:0043547)*, *Nervous system development (GO:0007399)*, *Regulation of transcription from RNA polymerase II promoter (GO:0006357)*, *Covalent chromatin modification (GO:0016569).*

Due to the high level of similarity in pathways of predicted target mRNAs at both ages, target genes were also predicted for the uniquely up-regulated and uniquely down-regulated miRNAs at each age. To do this, miRNAs with the same direction of change between fetuses and adolescent sheep were removed and gene target prediction was repeated. This method was used to identify pathways that were differentially regulated as a function of age in response to infarction (Tables 2, 3). Predicted mRNAs of the uniquely deregulated miRNAs that were oppositely expressed in fetuses and adolescent sheep were significantly enriched for *FoxO signaling pathway (map04068), Proteoglycans in cancer (map05205)* and *Neurotrophin signaling pathway (map04722)* KEGG terms (Table 2). *Insulin signaling pathway (map04910)* and *Sphingolipid signaling pathway (map04071)* were uniquely enriched for the predicted target genes of up-regulated and down-regulated miRNAs, respectively, in the fetuses (Table 2). *Rap1 signaling pathway (map04015)* and *Fc gamma R (map04666)* were both uniquely enriched for the predicted target genes of up-regulated miRNAs whereas *Wnt signaling pathway (map04310)* was enriched for the predicted target genes of down-regulated miRNAs in the adolescent Infarct samples (Table 2). Uniquely enriched gene ontology terms for Biological Processes included *Canonical Wnt signaling pathway (GO:0060070)* and *Endocytosis (GO:0006897)*, which were oppositely regulated at each age (Table 3). *Wnt signaling pathway (GO:0016055)* was uniquely enriched for the predicted target genes of up-regulated miRNAs in the fetuses (Table 3). Whereas *Collagen fibril organization (GO:0030199), Intracellular protein transport (GO:0006886)*, and *Protein localization to plasma membrane (GO:0072659)* were uniquely enriched in the adolescent sheep.

**TABLE 2:** Top 10 KEGG pathway enrichment terms for predicted targets of uniquely deregulated miRNAs in the infarct compared to remote samples.

**Treatment group**	**KEGG pathway terms**	**Term accession**	**Pathway hits**	**P^adj^**
Predicted targets of fetal up-regulated miRNAs	Axon guidance	map04360	98	0.000
	Pathways in cancer	map05200	180	0.000
	FoxO signaling pathway	map04068	73	0.0091
	Proteoglycans in cancer	map05205	101	0.0091
	MAPK signaling pathway	map04010	119	0.0091
	Insulin signaling pathway	map04910	75	0.0091
	ErbB signaling pathway	map04012	51	0.0102
	Ras signaling pathway	map04014	109	0.0102
	AMPK signaling pathway	map04152	68	0.0109
	Regulation of actin cytoskeleton	map04810	103	0.0109
Predicted targets of fetal down-regulated miRNAs	Axon guidance	map04360	119	0.000
	Ras signaling pathway	map04014	142	0.000
	Pathways in cancer	map05200	229	0.000
	AMPK signaling pathway	map04152	87	0.007
	Oxytocin signaling pathway	map04921	101	0.0081
	ErbB signaling pathway	map04012	64	0.0081
	Neurotrophin signaling pathway	map04722	82	0.0081
	cAMP signaling pathway	map04024	119	0.0106
	MAPK signaling pathway	map04010	145	0.0113
	Sphingolipid signaling pathway	map04071	82	0.0113
Predicted targets of adolescent up-regulated miRNAs	Endocytosis	map04144	123	0.000
	Axon guidance	map04360	81	0.0055
	Ras signaling pathway	map04014	98	0.0055
	Regulation of actin cytoskeleton	map04810	95	0.0055
	Neurotrophin signaling pathway	map04722	62	0.0055
	AMPK signaling pathway	map04152	62	0.0069
	Rap1 signaling pathway	map04015	91	0.0069
	Fc gamma R	map04666	47	0.0069
	Pathways in cancer	map05200	150	0.0082
	cAMP signaling pathway	map04024	85	0.0082
Predicted targets of adolescent down-regulated miRNAs	MAPK signaling pathway	map04010	169	0.000
	Axon guidance	map04360	127	0.000
	Wnt signaling pathway	map04310	106	0.000
	Pathways in cancer	map05200	258	0.000
	Proteoglycans in cancer	map05205	136	0.0041
	FoxO signaling pathway	map04068	98	0.0041
	Ras signaling pathway	map04014	152	0.0041
	cAMP signaling pathway	map04024	130	0.0072
	Oxytocin signaling pathway	map04921	107	0.0078
	ErbB signaling pathway	map04012	67	0.0078

**P-values were corrected for multiple testing using Benjamini-Hochberg method. P^*adj*^ is the FDR q-value. All enrichments were performed through miRWalk.**

**TABLE 3:** Top 10 gene ontology biological process terms for predicted mRNA targets of uniquely deregulated miRNAs.

**Treatment group**	**Biological processes**	**Term accession**	**Pathway hits**	**P^adj^**
Predicted targets of fetal up-regulated miRNAs	Protein ubiquitination	GO:0016567	153	0.0001
	Response to ATP	GO:0033198	38	0.0003
	Peptidyl-serine phosphorylation	GO:0018105	114	0.0007
	Adenosine catabolic process	GO:0006154	23	0.0007
	Intracellular signal transduction	GO:0035556	213	0.0007
	Protein dephosphorylation	GO:0006470	98	0.0011
	Cellular response to heat	GO:0034605	39	0.0013
	Positive regulation of NF-kappaB transcription factor activity	GO:0051092	88	0.0014
	Canonical Wnt signaling pathway	GO:0060070	111	0.0015
	Wnt signaling pathway	GO:0016055	127	0.0017
Predicted targets of fetal down-regulated miRNAs	Water transport	GO:0006833	44	0.0008
	Positive regulation of MAPK cascade	GO:0043410	83	0.0011
	Peptidyl-serine phosphorylation	GO:0018105	139	0.0014
	Intracellular signal transduction	GO:0035556	262	0.0016
	Endocytosis	GO:0006897	131	0.0035
	Protein ubiquitination	GO:0016567	174	0.0037
	Peptidyl-tyrosine dephosphorylation	GO:0035335	44	0.0039
	Response to ATP	GO:0033198	40	0.0041
	Protein polyubiquitination	GO:0000209	89	0.0045
	Actin cytoskeleton organization	GO:0030036	104	0.0052
Predicted targets of adolescent up-regulated miRNAs	Nervous system development	GO:0007399	192	0.0001
	Intracellular signal transduction	GO:0035556	193	0.0002
	Sensory perception of sound	GO:0007605	107	0.0006
	Neuron migration	GO:0001764	90	0.0008
	Collagen fibril organization	GO:0030199	44	0.001
	Endocytosis	GO:0006897	97	0.0011
	Intracellular protein transport	GO:0006886	107	0.0012
	Positive regulation of I-kappaB kinase/NF-kappaB signaling	GO:0043123	83	0.0015
	Memory	GO:0007613	54	0.0016
	Peptidyl-serine phosphorylation	GO:0018105	98	0.0018
Predicted targets of adolescent down-regulated miRNAs	Neuron migration	GO:0001764	141	0.0001
	Peptidyl-serine phosphorylation	GO:0018105	153	0.0004
	Intracellular signal transduction	GO:0035556	288	0.0004
	Protein autophosphorylation	GO:0046777	175	0.0011
	Protein localization to plasma membrane	GO:0072659	117	0.0012
	Canonical Wnt signaling pathway	GO:0060070	147	0.0015
	Nervous system development	GO:0007399	134	0.0015
	cAMP catabolic process	GO:0006198	21	0.0015
	Protein ubiquitination	GO:0016567	189	0.0019
	Actin cytoskeleton organization	GO:0030036	59	0.0025

**P-values were corrected for multiple testing using Benjamini-Hochberg method. P^*adj*^ is the FDR q-value. All enrichments were performed through miRWalk.**

### Identification of Potential miRNA Targets for Treatment of Cardiac Disease

**FIGURE 5:**
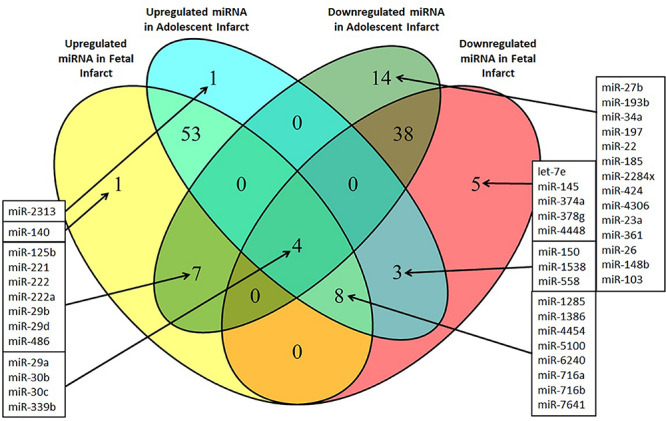
Venn diagram of differentially expressed miRNAs in the Infarct samples compared to Remote samples and Border samples in fetal and adolescent sheep. *n* = 3 per age group. Only miRNAs that were significantly upregulated or downregulated are shown (*P* < *0.05).*

miRNA responses to infarction at one age, but not the other may potentially contribute to the differing physiological responses to infarction at the fetal and adolescent ages, and therefore are of particular interest. These specific miRNA responses may underpin the differing repair capacities of fetal and adolescent sheep heart tissues. Several miRNAs were detected that had opposite expression profiles in fetus compared with adolescent sheep (Figure 5). miR-140 was uniquely up-regulated in fetal Infarct compared to Remote samples. This miRNA has a role in cardiac muscle hypertrophy (Joshi et al., 2016) as well as mitochondrial fission and apoptosis through mitofusin 1 (Mfn1) in rat hearts (Li et al., 2014). Five miRNAs were significantly down-regulated in the fetal Infarct compared to the Remote samples (let-7e, miR-145, miR-374a, miR-378g, and miR-4448; Figure 5). Some of these miRNAs were previously investigated including miR-145, which protects the heart from autophagy after infarction in rabbits (Higashi et al., 2015), but hypoxia downregulates the expression of this miRNA in mouse cardiac fibroblasts (Wang et al., 2014). miR-374 regulates vascular endothelial growth factor receptor-1 signaling in rats and modulates the inflammatory process in humans (Lee et al., 2017; Doumatey et al., 2018). 14 miRNAs were uniquely down-regulated in the adolescent Infarct compared to Remote samples (Figure 5) and included some miRNAs known to be involved in rodent cardiac regeneration, including miR-34a (a miRNA associated with cardiac repair in mice; Yang et al., 2015) and miR-26, which plays a major role in regulating cardiac collagen I expression in rats (Zheng et al., 2018). Inhibition of the miR-34 family has been shown to have therapeutic potential for human cardiac pressure overload and MI (Bernardo et al., 2012; Boon et al., 2013; Lock et al., 2017). The expression of the miR-34 family is increased in the mouse heart after MI (Lin et al., 2010) and in cardiac tissue from patients with heart disease (Thum et al., 2007; Greco et al., 2012). In sheep heart tissue we observed the opposite expression profile, with down-regulation of miR-34a in Infarct compared to Remote samples in adolescent sheep and no change in fetal expression. This result indicates that the miR-34 family may have a less prominent role in sheep heart responses to infarction compared to other mammals. One miRNA (miR-2313) was uniquely up-regulated in the adolescent Infarct samples compared to Remote samples. This miRNA was discovered in the bovine genome, and possible human homologs, which may represent a potential target for inhibition after infarction. Since the microarray contained probes from multiple species for the same miRNA, four miRNAs appeared to be both up-regulated and down-regulated in both the fetuses and lambs, this was an artifact of the multispecies redundancy likely due to slightly different seed sequences between species leading to inconsistent binding of the miRNAs to the probes. Since the direction of change was inconclusive, these miRNAs were not investigated further. Several miRNAs were oppositely regulated in each age group. Of particular interest were three miRNAs that were significantly up-regulated in the adolescent Infarct, but were down-regulated in the fetal Infarct (miR-1538, miR-558, and miR-150). miR-150 was previously investigated for its role in cancer metastasis (Wang et al., 2015; Li et al., 2016; Sun et al., 2016), pulmonary hypertension (Li et al., 2019) and B cell development (Zhou et al., 2007). mRNA targets of these three miRNAs (miR-150, miR-558, and miR-1538) were predicted using miRWalk, and gene ontology was performed to determine if the miRNAs may be involved in regulating functions that could be important in cardiac repair. Given that miRNAs have hundreds of potential target genes, identifying target pathways rather than specific genes allows a better overview of the potential role of miRNAs. KEGG pathway terms for predicted target mRNAs of miR-558 included *AMPK Signaling pathway* (*P* = 4.5E-07), *Axon guidance* (*P* = 3.97E-06), *Pathways in cancer* (*P* = 8.93E-05), *PI3K-Akt signaling pathway* (*P* = 0.0014) and *HIF-1 Signaling pathway* (*P* = 0.004). KEGG pathway terms for predicted target mRNAs of miR-1538 included *Circadian entrainment* (*P* = 7.63E-08), *ErbB signaling pathway* (*P* = 6.60E-08), *VEGF signaling pathway* (*P* = 1.30E-04), and several “Cancer” pathways synonymous with cell proliferation.

### H9c2 Cell Culture Analysis

**FIGURE 6:**
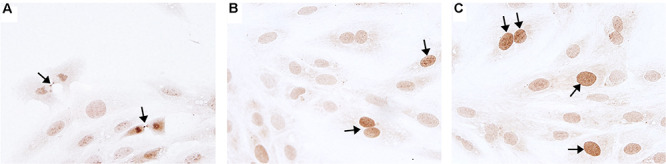
Aurora B immunohistochemistry staining. Representative micrographs of Aurora B immunohistochemistry staining (marker of cells entering cytokinesis) of H9c2 cardiomyoblasts after treatment with Hypoxia (1% O_2_). **(A)** Example of midbody staining in cells undergoing cytokinesis. **(B)** Negative control miRNA. **(C)** miR-558 inhibitor. Arrows indicate positive staining.

**FIGURE 7:**
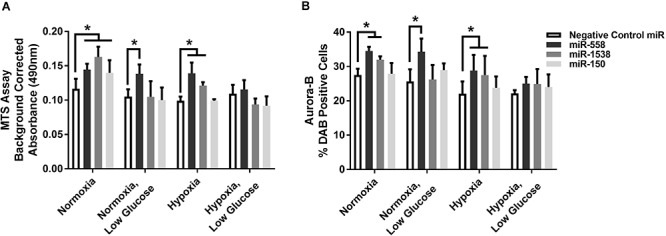
Cell proliferation measures. **(A)** MTS cell proliferation assay. Background corrected proliferation assay absorbance of test anti-miRNAs. **(B)** % DAB Positive cells stained using an anti-Aurora-B antibody in 80 counting frames (Negative Control anti-miRNA, anti-miR-558, anti-miR-1538, anti-miR-150) on H9c2 cardiomyoblasts under Normoxia/Hypoxia or Glucose/Low Glucose conditions. *Represents significantly different data from the Negative Control miR group (*P* < 0.05). Nx, normoxia; LG, low glucose; Hx, hypoxia. Mean ± Standard Deviation.

**FIGURE 8:**
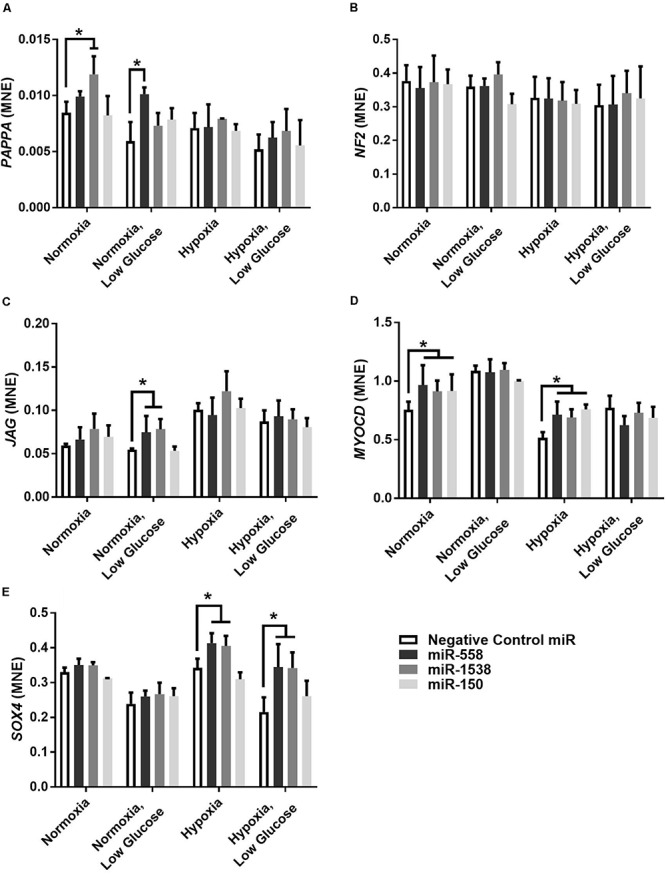
Target gene expression of anti-miRNAs. Mean normalized expression (MNE) of predicted target genes of anti-miRNAs, *PAPPA* **(A)**, *NF2* **(B)**, *JAG* **(C)**, *MYOC1* **(D)**, and *SOX4* **(E)** demonstrating successful inhibition of miRNA resulting in decreased inhibition of target mRNAs. *Represents significantly different data from the negative control group (*P* < 0.05). *n* = 4 per treatment group.

**FIGURE 9:**
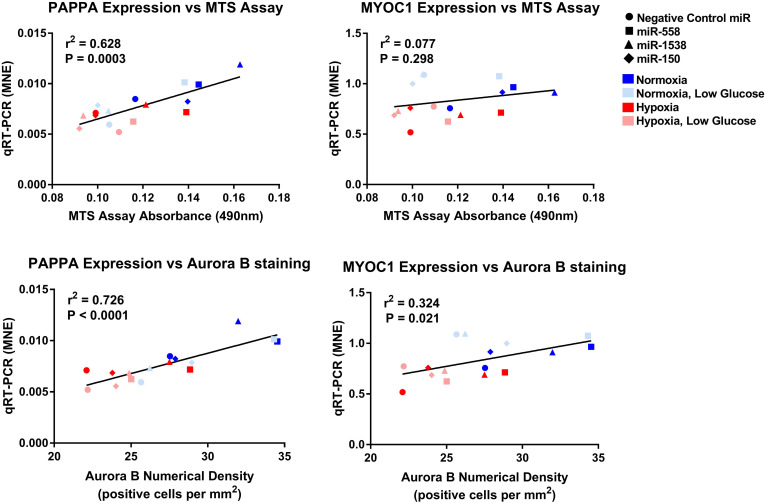
Linear regression of *PAPPA* and *MYOC1* expression against measures of proliferation. Mean normalized expression of predicted target genes of anti-miRNAs against MTS and Aurora B proliferation measures demonstrating effectiveness of miRNA treatments within each oxygen environment.

Novel miRNA targets for treatment of cardiovascular disease were identified from the microarray data; these included miR-150, miR-558, and miR-1538, which were investigated further by their inhibition in cell culture. H9c2 cardiomyoblasts were cultured with anti-miRNAs for three miRNAs of interest (miR-558, miR-1538, and miR-150) as well as a negative control for a miRNA sequence not expressed in mammals (NC5; Integrated DNA Technologies, United States). Cell proliferation was measured in live and fixed cells using a colorimetric MTS assay and anti-Aurora-B (a marker of cells entering cytokinesis) immunohistochemistry staining, respectively. miR-558 inhibition caused an increase (*P* < 0.05) in cardiomyoblast proliferation under the “Normoxia,” “Normoxia, Low Glucose,” and “Hypoxia” conditions using both the MTS assay and Aurora-B staining (Figures 6, 7). miR-1538 inhibition caused an increase (*P* < 0.05) in cell proliferation under “Normoxia,” and “Hypoxia” conditions using both MTS assay and Aurora-B staining. miR-150 inhibition was least effective in promoting proliferation, only increasing cell proliferation (*P* < 0.05) using the MTS assay in “Normoxia.” The expression of predicted target mRNAs for miR-558 and miR-1538 included *PAPPA, JAG, NF2, MYOC1*, and *SOX4*. *NF2* was unchanged in response to miRNA inhibition (Figure 8). This could be due to a false-positive in the mRNA target prediction. miR-558 inhibition resulted in an increase in expression of *PAPPA* in “Normoxia, Low Glucose,” *MYOC1* in “Normoxia” and “Hypoxia,” *JAG* in “Normoxia, Low Glucose,” and *SOX4* in the “Hypoxia” and “Hypoxia, Low Glucose” conditions (Figures 8, 9; *P* < 0.05). miR-1538 inhibition resulted in an increase in expression of *PAPPA* in “Normoxia,” *MYOC1* in “Normoxia” and “Hypoxia,” *JAG* in “Normoxia, Low Glucose,” and *SOX4* in the “Hypoxia” and “Hypoxia, Low Glucose” conditions (Figures 8, 9; *P* < 0.05). miR-150 inhibition increased the expression of only *MYOC1* in “Normoxia” and “Hypoxia” conditions (Figures 8, 9; *P* < 0.05). Overall, inhibition miR-558 and miR-1538 were effective for promoting cell proliferation *in vitro* and effectively modulating the expression of their predicted target mRNAs. Further mRNA targets may be identified from the differentially regulated miRNAs identified in the current investigation, and additional testing of these miRNAs on adult differentiated primary cardiomyocytes may provide evidence supporting enhanced cardiomyocyte proliferation in large animals at this age.

## Discussion

The predicted target mRNA term enrichments for miRNAs that were up-regulated or down-regulated in Infarct compared to Remote samples included a number of biological themes that could be associated with cardiac repair and regeneration. Common themes comprised cancer-related terms (may reflect increased cell proliferation), inflammatory cell/leukocyte migration (inflammation), protein transport and ubiquitination, MAPK signaling, and WNT signaling. Since it was not possible to perform target prediction against a cardiac-only background in miRWalk, there was an absence of muscle related enrichment terms. A number of these themes were present at both ages in response to infarction, indicating that a large number of pathways are similarly regulated in the regenerative and quiescent heart. Recent evidence by our group and others demonstrated a largely similar but highly attenuated gene expression response to infarction in the regenerative heart compared to the quiescent heart (Zgheib et al., 2014; Mills et al., 2017; Quaife-Ryan et al., 2017; Lock et al., 2019). This response indicates a “resistance” to damage in the regenerative heart and is supported by the similar enrichment terms of predicted targets from deregulated miRNAs after infarction, as demonstrated in this study (Supplementary File 2). In addition, some similar themes between fetal and adolescent sheep may be due to the nature of miRNAs targeting a number of mRNAs, false positives in the mRNA target prediction method, as well as gene pathway enrichments including both promotors and repressors of pathways. These factors may lead to a large number of similar pathway enrichments at both ages. Limiting the term enrichments to miRNAs that were exclusively up-regulated or down-regulated at each age helped tease apart the molecular mechanisms underpinning the proliferative capacity or lack thereof in fetuses and adolescent hearts, respectively. Some of the pathways that are targeted by the identified uniquely deregulated miRNAs within this study are discussed below and how they may be exploited to improve cardiac regeneration.

### Uniquely Deregulated Term Enrichments

#### FoxO Signaling

Forkhead box (Fox) family transcription factors have been implicated in cardiomyocyte cell cycle control as well as adult cardiovascular disease (Ronnebaum and Patterson, 2010). FoxO signaling was predicted to be oppositely regulated in the fetuses and adolescent sheep based on the miRNA target genes. miRNAs that suppress *FoxO* mRNA were up-regulated in the fetuses resulting in a decrease in FoxO expression in response to infarction, compared with an opposing decrease in miRNA expression in the adolescent sheep consistent with an increase in FoxO expression. Decreased FoxO activity in the proliferative fetal heart is a logical outcome given that FoxO is a major negative regulator of cardiomyocyte proliferation in fetal life. Forced expression of FoxO inhibits rat cardiomyocyte cell proliferation and induces expression of cell cycle inhibitors p21 and p27 (Sengupta et al., 2013). In the adult rat heart, activation of FoxO promotes cardiomyocyte survival under conditions of oxidative stress and is linked to AMPK and IGF signaling (Ronnebaum and Patterson, 2010), as well as many other pathways that were also significantly enriched in this study (*endoplasmic reticulum stress, autophagy, mitochondrial fission*, and *cell cycle*). FoxO therefore represents an interesting intermediary target pathway for miRNA inhibition to improve the repair response after infarction. However, there are a number of challenges associated with targeting specific isoforms of FoxO (FOXO1 and FOXO3) as well as differing effects within different cell types of the heart (Sengupta et al., 2013; Li et al., 2016). This would likely be an ongoing issue when utilizing miRNAs to target expression of this pathway as there would be probable cross-over suppression of both isoforms of FOXO as indicated by the predicted target mRNAs (from the fetal up-regulated miRNAs; miR-6337, miR-1197, miR-125b, miR-24, miR-1, miR-1285, miR-140, miR-221, miR-222, and miR-411b all have predicted targets in the FoxO pathway).

#### Wnt Signaling Pathway

In the current investigation, the Wnt Signaling and Canonical Wnt signaling pathways were both consistently regulated pathways by a large number of the differentially regulated miRNAs. The Wnt pathway has been an area of interest in recent years for cardiac disease due to its demonstrated role in the regeneration of cardiac tissue (and other organs such as bone marrow, intestines and skin) in small animal models (Lock et al., 2018). The data from the current investigation and other data suggests that this may be one of the pivotal pathways being regulated during cardiac repair and regeneration (Mills et al., 2017; Quaife-Ryan et al., 2017). Three Wnt signaling pathways have been characterized: (i) the canonical Wnt pathway, (ii) the non-canonical planar cell polarity (PCP) pathway, and (iii) the non-canonical Wnt/calcium pathway. These pathways each have differing transcription factors and target genes but are linked by the binding of a Wnt-protein ligand to a Frizzled family receptor on the plasma membrane, which interacts with the intracellular Disheveled protein. Wnt target genes are modulated through a number of mechanisms, with the central Wnt signaling proteins linking multiple important molecular pathways in cardiomyocyte proliferation and survival such as the Hippo and MAPK pathways (Lock et al., 2018). Of particular interest were miR-6337, miR-1197, miR-24, miR-25, miR-125b, miR-29b, miR-1285, miR-486, and miR-222, all of which were up-regulated in the fetal Infarct compared to Remote samples and had predicted targets in the Wnt signaling pathways. Further investigation of the specific deregulated miRNAs that target this pathway could cause transient modulation of this powerful signaling cascade and potentially alter the proliferative ability of cardiomyocytes after infarction.

#### Sphingolipid Signaling

Sphingolipid signaling pathway was significantly enriched for a number of the deregulated miRNAs. Sphingolipids are derivatives of the amino alcohol sphingosine and are active components of the cardiomyocytes cell membrane, which play an important role in intracellular signal transduction and regulate diverse cellular processes such as proliferation, maturation, apoptosis and the cellular stress response (Borodzicz et al., 2015). The most important sphingolipids include ceramide (CER), sphingosine (SPH), and sphingosine-1-phosphate (S1P). Some proteins such as tumor necrosis factor-α [TNF-α; which was down-regulated in the fetal Infarct samples compared to Remote samples and up-regulated in the adolescent Infarct samples compared to Remote and Sham samples (Lock et al., 2019)] induce synthesis of CER from sphingomyelin via sphingomyelinase. CER can then act as a second messenger, promoting the apoptosis of cardiomyocytes (Borodzicz et al., 2015). On the other hand, sphingosine-1-phosphate is cardioprotective (Jin et al., 2002; Knapp et al., 2012a; Karliner, 2013). The ratio of CER and S1P is particularly important for control of apoptosis of cardiomyocytes in the remote area of the myocardium after MI (Knapp et al., 2012b). miRNA regulation of the proteins involved in the synthesis and signaling cascade of sphingolipids appears to be important for their regulation following MI. In particular, let-7e, miR-6391, miR-378g, miR-4454, miR-671, miR-145, miR-574, miR-2411, miR-558, and miR-1538 were all down-regulated in the fetal Infarct compared to Remote samples and contained predicted mRNA targets within the sphingolipid signaling pathway. Further investigation of this signaling pathway may therefore allow for control of apoptosis in the non-infarcted areas of the heart after heart attack, mitigating cardiomyocyte loss and subsequent pathological hypertrophy.

#### Neurotrophin Signaling

The neurotrophin pathway was also significantly enriched for predicted mRNA targets of oppositely regulated miRNAs at both ages. Interestingly, brain derived neurotrophic factor (BDNF) has a cardioprotective role in the heart after infarction by preventing adverse remodeling, and acts on endothelial cells to promote neovascularization in response to hypoxic stimuli via the Akt pathway (Okada et al., 2012). From the miRNAs that were down-regulated in the fetus and were up-regulated in the adolescent sheep; let-7e and miR-6391 were of particular interest in the regulation of the neurotrophin signaling pathway as they had a large number of predicted target genes within this pathway (Supplementary File 2). Inhibition of these miRNAs may therefore help prevent adverse tissue remodeling after heart attack.

#### Cardiac Fibrosis

Regulation of cardiac fibrosis and extracellular matrix deposition following infarction is crucial to maintain contractile function of the ventricles and prevent ventricular rupture (Sattler and Rosenthal, 2016). We previously reported increased percentage of staining of picrosirius red in the Infarcted samples compared with Sham samples for both age groups (Lock et al., 2019), but that the localization of the staining was different between the two age groups (the fetal sheep had larger deposition of collagen in the Border-zone than the Infarct area). *COL1A1* is the major component of type 1 collagen forming a large portion of the extracellular matrix (Pauschinger et al., 1998). We have also previously reported that there was an opposite expression profile between fetuses and adolescent sheep for both the collagen genes *COL1A1* and *COL3A1* between the individual tissue regions, indicating a reciprocal relationship between the two collagen types in response to infarction (Lock et al., 2019). This difference of collagen type following infarction is important as these collagen fibers maintain different elasticity, stiffness and capacity for degradation by metalloproteinases (MMPs) and is therefore likely one of the aspects of the fetal heart that allows for its regeneration. Although this scar formation allows for higher survival rates after infarction, the diminished contractile ability of the scar tissue has detrimental effects on long term heart function leading to chronic heart disease (AIHW, 2015). Interestingly, there was an up-regulation of miRNAs in the adolescent Infarct samples that had predicted mRNA targets involved in collagen fibril organization (miR-25, miR-3535, miR-6391) indicating a down-regulation of collagen organization within the adolescent Infarct samples. Another miRNA of interest was miR-26, which was down-regulated in the adolescent Infarct samples and plays a major role in the regulation of cardiac collagen I expression (Zheng et al., 2018). The observed decrease in miR-26 expression may help explain the increased expression of *COL1A1* in the adolescent Infarct samples and represents a valuable molecular target for up-regulation to prevent adverse cardiac fibrosis and reduce infarct size post-infarct setting in adults.

### Potential Targets for miRNA Therapeutic Inhibition

The current investigation identified 10 miRNAs that were oppositely regulated in fetuses and adolescent sheep and the effect of inhibition of these miRNAs on cardiomyocyte proliferation was then assessed *in vitro.* Inhibiting miR-558 and miR-1538 was effective for increasing the expression of their target mRNAs and increasing the number of cells within the cell cycle in H9c2 cultured cells. SOX4 was only increased in the hypoxia anti-miRNA treatment groups, which is likely due to hypoxia-inducible factors (*HIF1A* and *HIF3A*) being amongst the predicted targets for miR-558 and miR-1538. SOX4 most likely has a hypoxia response element within its promotor region, resulting in an up-regulation in gene expression only in the hypoxia anti-miRNA treatment groups (Yeh et al., 2013). One limitation of this cell culture approach is that the anti-miRNA treatments were tested in an immortal cardiomyoblast cell line, which already has a high rate of proliferation. Through this study, we identified unique miRNAs in sheep and were able to successfully inhibit these miRNAs in rat cardiomyoblasts, thereby demonstrating the potential translational capacity of miRNAs as a treatment between species. Further studies inhibiting these miRNAs *in vivo* will determine if this treatment has the capacity to improve the cardiovascular outcomes and reduce scar tissue formation after myocardial infarction, and may prove to be a powerful clinical intervention for transient changes in gene expression helping prevent the onset of chronic heart disease.

## Conclusion

Through investigating cardiac regeneration in fetal sheep, we have identified potential miRNA therapeutics for cardiovascular disease. Though the translational capacity and high throughput benefits of small animal experiments is clear, further investigation of therapeutics using large animal models, such as sheep and pigs, will help bridge the physiological gap, and allows for identification of additional unique targets for investigation. Delivery of miRNAs specifically to the heart remains a significant challenge for translation to clinical use, some studies have demonstrated success with intravenous delivery; however, to avoid potential off-target effects, intracardiac delivery is the current preferred *in vivo* delivery method. Although it was not possible to assess subtle sex specific effects within this study, our data suggests no effect of sex. This is apparent in the PCA plot where the fetal Remote samples were virtually super-imposable and contained two male and one female sample. In addition, our previous studies using this model demonstrated no significant difference between sex for sham or remote samples using gene array (Lock et al., 2020). However, we recognize that evaluating the response to infarction in both sexes is clinically important and will be assessed in future studies. A powerful tool in the study was the inclusion of the salvageable border zone tissue. This tissue is the first site in which cardiomyocyte proliferation begins in mammals capable of regeneration (Herdrich et al., 2010; Porrello et al., 2011; Zgheib et al., 2014), and thus important changes within this region may be missed in studies measuring expression on only the infarct and healthy tissue regions. Predicted mRNA targets of deregulated miRNAs were most significantly enriched for *FoxO, Wnt*, and *Cardiac Fibrosis* signaling after infarction in the sheep heart. miRNAs and their targets are often conserved between species, hence targeting these pathways through modulation of identified uniquely deregulated miRNAs may improve repair of the adult human heart. miR-558 and miR-1538 were oppositely regulated in the fetal and adolescent hearts in response to MI. When inhibited in H9c2 cells these miRNAs were both effective targets for increasing cell proliferation and target gene expression and therefore represent strong prospective therapeutics to improve cardiac outcomes post-MI. The next step is to interrogate the efficacy of these two miRNAs using the same MI sheep model. These miRNAs may also be useful targets for cardiovascular diseases outside of MI where an increase in cardiomyocyte proliferation would be beneficial. One such example is fetuses with intrauterine growth restriction (IUGR) that have a lower cardiomyocyte endowment than normally grown fetuses (Botting et al., 2014). The lower number of cardiomyocytes in IUGR infants leads to pathological hypertrophy and an increased risk of cardiovascular disease in later life (Wang et al., 2011; Lock et al., 2017). Modulating the expression of these miRNAs in damaged hearts allows for a short-term change in gene expression and may aid in prevention of the lasting development of chronic heart disease.

## Data Availability Statement

The microarray data in this study has been deposited into Figshare (https://figshare.com/s/27972b2c60aeb4cac853).

## Ethics Statement

The animal study was reviewed and approved by the South Australian Health and Medical Research Institute (SAHMRI) Animal Ethics Committee.

## Author Contributions

ML, DB, and JM were responsible for the conception and design of the experiments. ML, JYS, JD, MS, JBS, and JM were involved in experimentation and sample and data acquisition. ML and JM drafted the manuscript. All authors were involved in analysis, interpretation of the data, and contributed to the final version.

## Conflict of Interest

The authors declare that the research was conducted in the absence of any commercial or financial relationships that could be construed as a potential conflict of interest.

## Footnotes

1 http://www.mirbase.org/

2 http://www.clcbio.com/

3 https://biit.cs.ut.ee/clustvis/

4 http://mirwalk.umm.uni-heidelberg.de/
