# Supplementary material for: Identification of Novel miRNAs Involved in Cardiac Repair Following Infarction in Fetal and Adolescent Sheep Hearts
Source: Front Physiol. 2020 Jun 10;11:614. doi: 10.3389/fphys.2020.00614 (PMC7298149; doi:10.3389/fphys.2020.00614)
Supplement: Supplementary file 1 [file Table_1.DOCX]

**Supplementary Figure 1: qRT-PCR validation of miR-199a and miR-590 target mRNA expression** **in sheep three days post MI**. Mean normalised expression (MNE) of *CLIC5* (A), *HOPX* (B) and *HOMER1* (C) in Sham, Remote, Border zone, and Infarct Tissue*.* Superscript letters (Fetal Sheep; a, b and Adolescent Sheep; x, y) represent significance between tissue regions (Remote, Border and Infarct) at each age (*P*<0.05). * represents significantly different data from the sham animals at each age (*P*<0.05). Analyses between tissue regions (Infarct vs Border vs Remote) at each age were assessed using a nested Analysis of variance (ANOVA). A Bonferroni post-hoc test was performed with multiple comparisons for each tissue region against the Sham tissue. n=5 per treatment group per age.

**
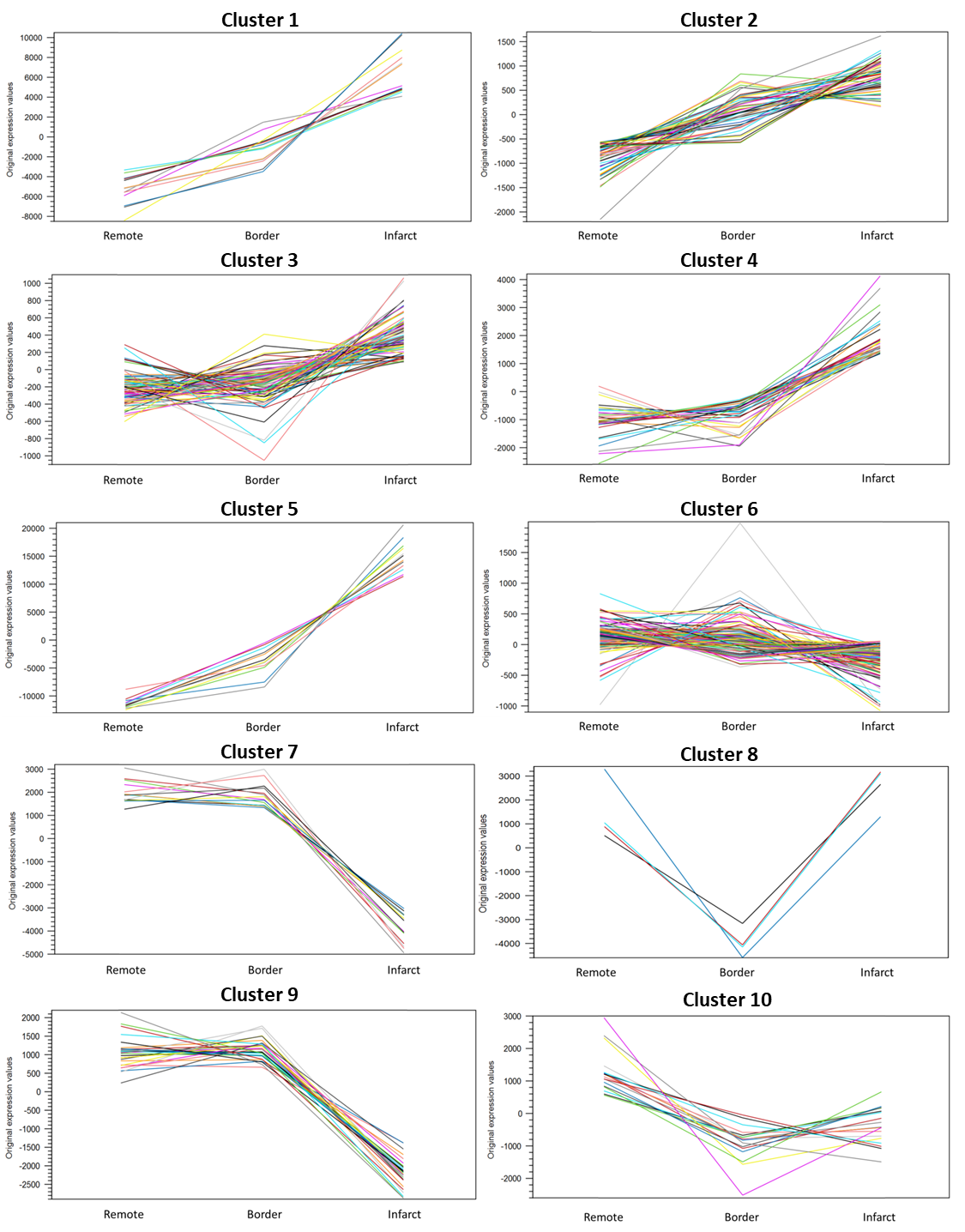
**

**Supplementary Figure 2:** Fetal sheep K-means clustering. Each coloured line represents a probe on the microarray that was differentially expressed in sheep three days after infarction.


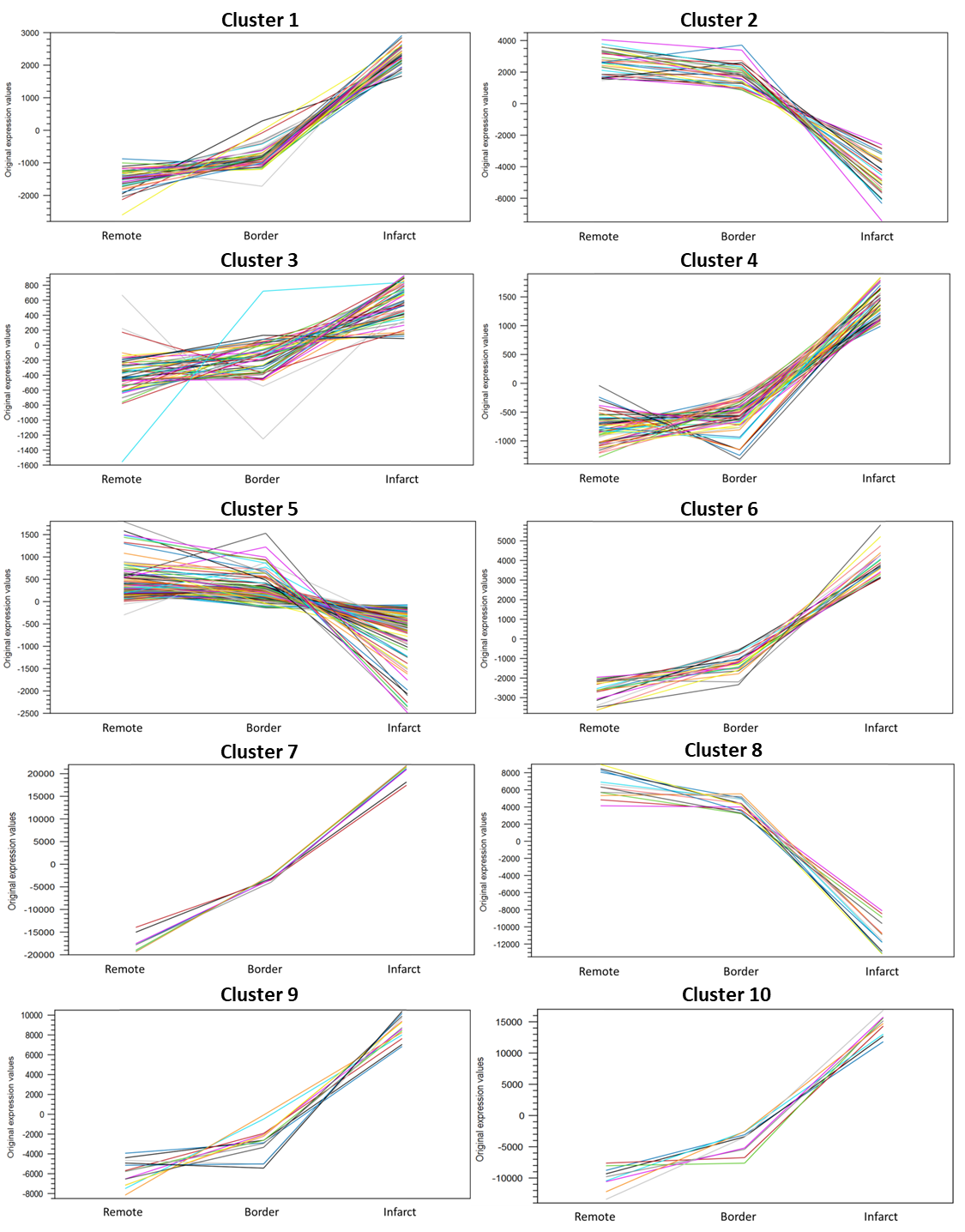


**Supplementary Figure 3:** Adolescent sheep K-means clustering. Each coloured line represents a probe on the microarray that was differentially expressed in sheep three days after infarction.

**Supplementary Figure 4: qRT-PCR and microarray fold change comparisons**. Normalised expression was scaled relative to the respective Remote samples.
